# Cardiovascular Magnetic Resonance Imaging of Scar Development Following Pulmonary Vein Isolation: A Prospective Study

**DOI:** 10.1371/journal.pone.0104844

**Published:** 2014-09-24

**Authors:** Jeff Hsing, Dana C. Peters, Benjamin R. Knowles, Warren J. Manning, Mark E. Josephson

**Affiliations:** 1 Department of Medicine, Cardiovascular Division, Beth Israel Deaconess Medical Center, Harvard Medical School, Boston, Massachusetts, United States of America; 2 Department of Radiology, Beth Israel Deaconess Medical Center, Harvard Medical School, Boston, Massachusetts, United States of America; 3 Department of Radiology, Yale Medical School, New Haven, Connecticut, United States of America; University of Giessen Lung Center, Germany

## Abstract

**Aims:**

Cardiovascular magnetic resonance (MR) provides non-invasive assessment of early (24-hour) edema and injury following pulmonary vein isolation (by ablation) and subsequent scar formation. We hypothesize that 24-hours after ablation, cardiovascular MR would demonstrate a pattern of edema and injury due to ablation and the severity would correlate with subsequent scar.

**Methods:**

Fifteen atrial fibrillation patients underwent cardiovascular MR prior to pulmonary vein isolation, 24-hours post (N = 11) and 30-days post (N = 7) ablation, with T2-weighted (T2W) and late gadolinium enhancement (LGE) imaging. Left atrial wall thickness, edema enhancement ratio and LGE enhancement were assessed at each time point. Volumes of LGE and edema enhancement were measured, and the circumferential presence of injury was assessed at 24-hours, including comparison with LGE enhancement at 30 days.

**Results:**

Left atrial wall thickness was increased 24-hours post-ablation (10.7±4.1 mm vs. 7.0±1.8 mm pre-PVI, p<0.05). T2W enhancement at 24-hours showed increased edema enhancement ratio (1.5±0.4 for post-ablation, vs. 0.9±0.2 pre-ablation, p<0.001). Edema and LGE volumes at 24-hours were correlated with 30-day LGE volume (R = 0.76, p = 0.04, and R = 0.74, p = 0.09, respectively). Using a 16 segment model for assessment, 24-hour T2W had sensitivity, specificity, and accuracy of 82%, 63%, and 79% respectively, for predicting 30-day LGE. 24-hour LGE had sensitivity, specificity, and accuracy of 91%, 47%, and 84%.

**Conclusions:**

Increased left atrial wall thickening and edema were characterized on cardiovascular MR early post-ablation, and found to correlate with 30-day LGE scar.

## Introduction

Atrial fibrillation (AF) is the most common sustained arrhythmia, with substantial associated morbidity and mortality [1]. Pulmonary vein isolation (PVI) has been advocated as a therapy for AF, with freedom from AF reported in 60% to 85% of patients with paroxysmal AF [2]. Recurrence also depends on follow-up duration [3].

A suspected cause of AF recurrence is incomplete circumferential PVI ablation with associated electrical reconnection of the PVs to the left atrium (LA) [4]–[6]. One hypothesis to explain early (<30 days) (but not late) isolation after PVI is that the PVI procedure causes early focal *reversible* edema without permanent injury, resulting in only temporary electrical isolation. After the edema resolves, the PVs are electrically reconnected. During the PVI procedure, the extent of injury that is created during PVI is unknown, as neither fluoroscopy nor electroanatomical mapping allows for discrimination of the ablation lesions.

Cardiovascular magnetic resonance (CMR) is a non-invasive imaging modality that allows for identification of myocardial scar [7], [8], and edema [9] using late gadolinium enhancement (LGE) and T2-weighted (T2w) imaging, respectively. Animal studies have shown a close correspondence between CMR and injury after RF ablation [10], [11].

LGE CMR is also able to identify LA and PV scar late (≥30 days) after ablation, showing a trend of more extensive scarring in subjects who later recur [12]–[14]. Further, in patients undergoing redo procedures, isolated PVs had greater amount of ostial scar by LGE (43%) vs. those which were reconnected (21%) [15]. Therefore, it may be valuable to acutely identify PVs which will exhibit insufficient chronic scarring. Recent CMR studies have examined and characterized the development of LGE after ablation, comparing early and later imaging findings [11]–[13], [16], [17]. A critical issue for the important goal of CMR-guided ablation—whether as adjunct to catheterization or ablation within the CMR suite [18]–[22] — is to identify early imaging findings that are predictive of late LA wall scar after PVI.

Acute ablation lesions may consist of inflammation, coagulation necrosis and hemorrhage. Acute post-PVI imaging findings include an increase in LA wall thickness, likely due to edema [23], [24]. Other findings include LGE enhancement or dark no-reflow regions (where contrast agent is completely excluded, also called microvascular obstruction (MVO) [25]) and enhancement on T2W imaging. Knowles et al. visualized acute LA wall edema after PVI in humans [16], and found that T2W evidence of edema was more wide spread than acute LGE enhancement. Acute LGE patterns have been compared to subsequent/late LGE patterns [17], showing a more widespread and less intense enhancement on early LGE, and the transition of MVO acutely to enhancement on subsequent LGE [26]. This has also been reported in animal studies of acute ablation, where MVO persists for more than 45 minutes [10], with acutely injured necrotic tissue very slowly enhancing.

Little is known regarding the relationship of early injury (i.e. edema/necrosis) with late LGE scarring (i.e. fibrosis). Late LGE imaging (1 to 3 months) after PVI demonstrates a pattern of scarring in the LA [12], which correlates with clinical outcomes and to ablation locations [27]–[29]. Evidence shows that at 1 month post-PVI, edema has resolved [23]. LGE patterns do not change from 3 to 6–9 months [17].

We hypothesized that early (24 hours) after PVI, the PVs and LA would demonstrate transient injury, characterized by CMR enhancement on T2W and LGE images and that some of these changes would correlate with 30-day LGE scar. We sought to replicate prior studies of the correlation between acute LGE and acute T2W imaging [16], and between acute and late LGE imaging [30]. Further we sought to add to the existing knowledge of the relationship of acute T2w to 30-day LGE.

## Methods

### Patients

**Figure 1:**
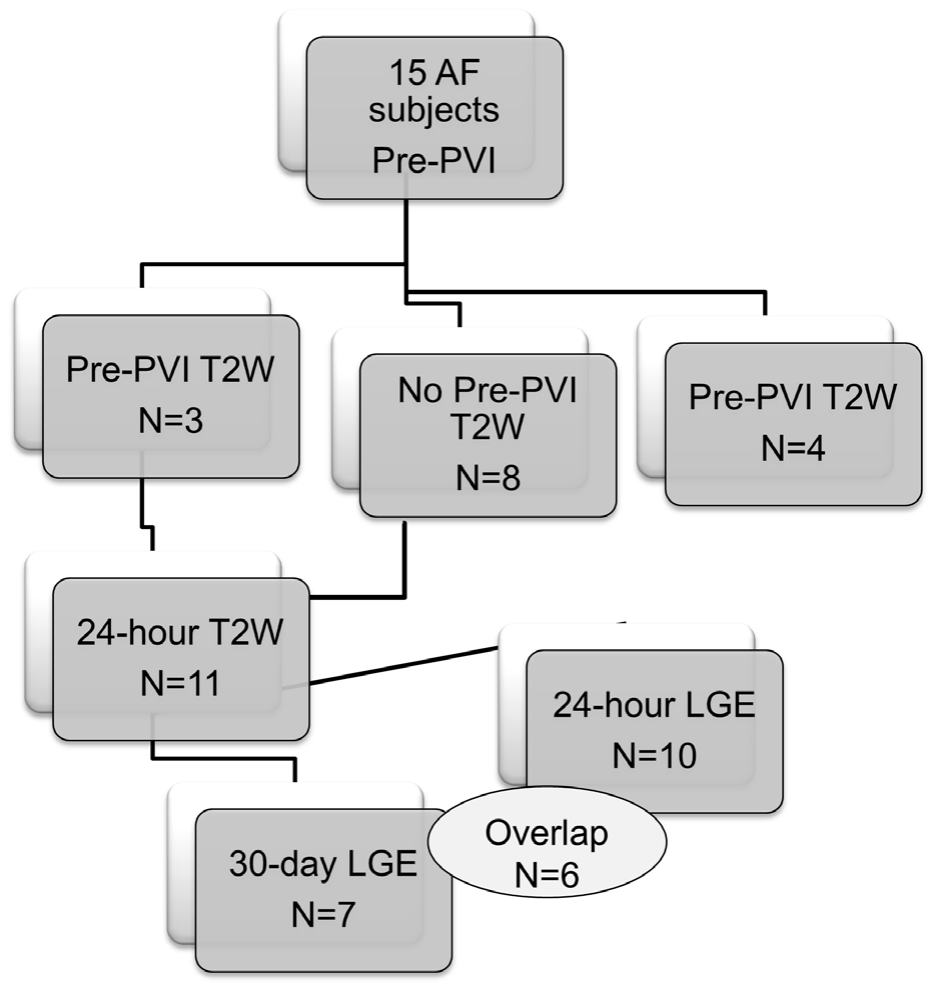
Flow chart describing the number of patients with T2W and LGE images at each time-point for the entire cohort. T2W = T2-weighted imaging. LGE = Late gadolinium enhancement. PVI = pulmonary vein isolation. Data from all available studies were used in all analyses.

Fifteen AF patients referred for their first PVI were prospectively studied, although not all images were acquired at each time-point (see Figure 1). Written informed consent for CMR was provided by all subjects and the study was approved by the Beth Israel Deaconess Medical Center Committee on Clinical Investigations. The study complies with the Declaration of Helsinki.

### Pulmonary Vein Isolation (PVI) Procedure

In brief, as described elsewhere [31], a 3.5 mm Biosense Thermocool (Biosense, Webster, Diamond Bar, CA, USA) irrigated catheter was advanced witin a long sheath into the LA, using intracardiac echocardiography guidance. A lasso catheter was advanced through a second long sheath and placed at each PV ostium for guiding the ablations and to confirming PV entrance and exit block. Radiofrequency (RF) ablation lesions were made 5–10 mm outside of each PV ostium in a circumferential fashion. Each PV was isolated. Maximal power was set at 30 W and 40°C and ablation lesions were generated using a maximum of 15–30 seconds at each site based on changes in the local electrogram. Electroanatomic mapping was performed using CARTO XP (Biosense Webster, Diamond Bar, California). The ablation duration was reduced to 10–15 seconds during ablation of the posterior LA wall and near the esophagus as identified by intracardiac echocardiography. The ablation goal was the loss of all PV potentials and failure to capture the atrium during pacing all bipolar poles of the lasso with a pulse width of 5 ms at 10 mA. Additional lines were only made if left sided atrial flutter could be induced. Posterior wall debulking and targeting of complex fractionated atrial electrograms were not performed. Intracardiac echocardiography was performed prior to and after PVI to confirm the absence of pericardial effusions. All PVI procedures were performed by a single operator (MEJ).

### CMR Imaging Protocol

CMR was performed on a Philips 1.5T CMR scanner (Achieva, Phillips Healthcare, Best, The Netherlands). Prior to contrast injection, ECG-triggered navigator-gated, fat-saturated, T2W 2D black blood fast spin echo imaging was performed in a stack of axial images covering the LA. Image parameters included: 300 mm FOV, 192×192 matrix (1.5×1.5 mm^2^ zero-filled to 0.6×0.6 in-plane resolution), 5 mm slice thickness, 33 echo train length, echo spacing 5.4 ms, 90° flip, TE = 60 ms, 1 average, 15 slices with no gaps, imaging in diastole. Coil-sensitivity correction was used on the T2W images. LGE imaging was performed ∼20 minutes after the injection of 0.2 mmol/kg Gd-DTPA (N = 6; Magnevist, Bayer Healthcare, Leverkusen, Germany; or 0.2 mmol/kg Gd-BOPTA (N = 1; MultiHance; Bracco, Princeton, NJ). The LGE sequence was an ECG-triggered navigator-gated 3D inversion recovery gradient echo sequence with fat saturation. Imaging parameters included: 320 mm FOV, 224×224 matrix, 4 mm slice thickness (spatial resolution 1.4×1.4×4 mm zero-filled to 0.6×0.6×2 mm^3^), TR/TE/θ = 5.3 ms/2.1 ms/25°, centric acquisition, with 100–150 ms acquisition window in diastole. The inversion time was set to null LV myocardium, using a Look-Locker sequence [32].

### Image Analyses

Image analyses were performed using ImageJ (NIH Image, Bethesda, MD) and 3D Slicer (v3.6, NA-MIC, Boston, MA).

### LA Wall Thickness

**Figure 2:**
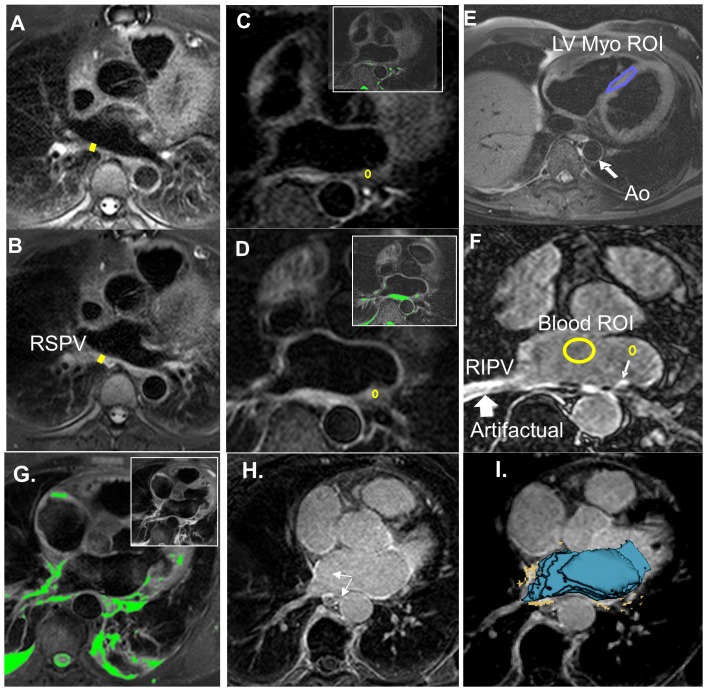
Methods of data analysis. A-B) T2W images from a subject imaged pre pulmonary vein isolation (PVI) (A) and 24-hour post PVI (B), used for measuring right superior PV wall thickness, as shown; note the prominent LA wall thickening. C-F) Another subject imaged pre-pulmonary vein isolation (PVI), 24-hour post-PVI, and 30-day post-PVI. C) Pre-PVI T2W image. D) 24-hours post -PVI T2W image shows enhancement where none is observed pre-PVI. Colored insets highlight regions where signal exceeds the edema threshold in C and D. ROIs were placed on the T2W images, as shown in (C, D). E) Axial T2-weighted (T2W) image at the level of the mid left ventricle (LV) with the region of interest (ROI) used for calculating the edema threshold (defined as 1.4 times the mean LV myocardium signal). F) Late gadolinium enhancement (LGE) 30-days post-PVI demonstrating scar. A left lower PV wall ROI, and blood pool ROI are shown. G) Color) Early post-PVI, the T2W image shows enhancement. Regions above the EER threshold (1.4) are highlighted in green. H) 30-day LGE appears to correlate 24-hour T2W. I) By registration of 24-hour T2W and 30-day LGE, the correlation between acute edema and later scar is more easily quantified, using matched ROIs. The blue shell represents the LA cavity segmented from the 30-day LGE images. The segmented edema (orange) is overlaid on the 30-day LGE image.

LA wall thickness was measured at one location pre- and post-PVI, in an unmatched cohort (see Figure 1). Posterior LA wall thickness measurements at the level of the right superior PV (RSPV) were made on T2W images by a blinded observer. The RSPV location (Figure 2A,B) was chosen to correspond to the location described by Okada et al. [23]. Thickness was measured by an experienced reader, on twice-zoomed images, with the line tool. Thickness measurement reproducibility was tested by repeating measurements (but not imaging) on a different day.

### Edema Enhancement Ratio (EER): T2_W_ Image Analysis Pre- vs. Post-PVI

To investigate the development of edema post-PVI, the signal in a small region-of-interest (ROI) of about 60 pixels containing the brightest region of each PV ostial wall was measured on T2W images by a blinded observer on pre- and 24-hour post-PVI images. The edema enhancement ratio (EER; Equation 1), the ratio of LA wall (Figure 2C, D) to LV myocardial signal (Figure 2E) was calculated, as previously described [33]. 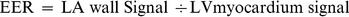
(Eq.1)


The EER threshold for edema was chosen as 1.4, based on pre-PVI EER measurements (mean +3SDS, see Results).

### CNR Comparison of 24-Hour vs. 30-Day LGE

In a similar fashion, the contrast-to-noise (CNR; scar to blood, Figure 2F) was measured, using a small region-of-interest (ROI) for scar, containing the most enhanced region of each PV ostial wall on LGE images. The measurement was performed by a blinded observer on 24-hour and 30-day post-PVI images (excluding data from the single patient who received Gd-BOPTA). Mean blood pool signal was measured, and the standard deviation of signal in air-space was used to estimate noise.

### Correlation between 24-Hour T2W and LGE Findings and 30-day LGE in the LA

#### CNR and EER comparison at matched locations

To compare signal on the 24-hour and 30-day post-PVI images, the 30-day post-PVI LGE images were registered to the 24-hour LGE images using 3D Slicer (v3.6, NA-MIC), with a 3D rigid registration based on mutual information; the resulting transformation also registered the 30-day LGE to 24-hour T2W images. After registration, signal intensity in a small ROI was measured in two locations each with and without LGE enhancement/scar on 30-day LGE. The small ROI containing PV ostia wall (see Figure 2F,D) was placed on all images, at well-matched locations. For the T2W images, the EER was calculated. For LGE images, the wall-blood CNR was calculated.

In order to assess the adequacy of contrast in the LGE images, and compare 30 day and 24 hour LGE contrast, valvular CNR was measured using an ROI in a visible mitral valve segment, vs. the blood signal.

#### Qualitative agreement of injury on 24-hour and 30-day images, in a 16 segment model

**Figure 3:**
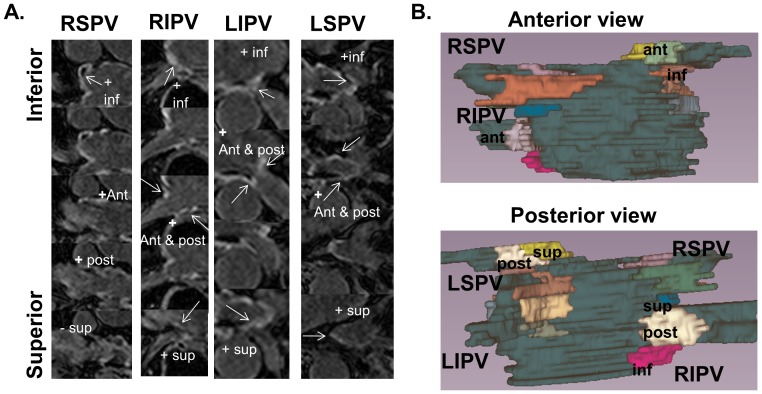
16 segment model showing the 4 regions evaluated around each pulmonary vein. A) Zoomed and cropped slices labeled by region. B) 3D color-coded display, showing 4 regions about each PV, with explicit labeling for the right inferior and left superior PV.

The presence of injury was assessed in a 16 segment model (Figure 3): 4 quadrants (anterior, posterior, inferior and superior) around each of 4 PVs. Blinded assessment was performed in all patients (4*4*7 = 112 total), on 24-hour T2W and LGE images, and 30-day LGE. These segments included PV wall territory within a radius ±1 cm from the ostia.

The presence of injury was assessed on T2W images, by using an EER>1.4. LGE was assessed visually, by noting enhancement having a CNR>∼3.5. A global threshold of CNR>3.5 SDs above the mean blood pool signal was used for LGE, based on a single mid-volume measurement (Figure 2F). This threshold choice has been demonstrated to optimally correlate with low voltage on post-ablation electroanatomic mapping [35]. When right PV ostial enhancement was apparent but adjacent to artifacts, the region was included as “injury” [34]. Since MVO has been shown to develop into scar [26], the presence of MVO was noted, and included as “injury”. For both T2w and LGE, any sizable enhanced territory (>3 mm diameter—excluding small islands) was classified as “injury” in that segment. The power of 24-hour T2W and LGE to predict late scar was studied using the 30-day LGE as the reference. Intra-observer variability was measured by repeating the qualitative analysis on a different day.

#### Quantitative volumetric comparison of 24-hour and 30-day injury

On all post-PVI T2W and LGE images, the entire LA cavity was traced, extending inferior to the most inferior PV, and superior to the LSPV, excluding the LA appendage, with care taken to exclude areas of artifactual enhancement (i.e. where enhanced wall is proximal to artifactually enhanced blood pool, Figure 2F). Using this tracing and a threshold of CNR>3.5, the volume of LGE enhancement was measured at 24-hours and 30-days. This semi-automated LGE segmentation method has been described in detail [36] and shown to outperform competing methods. For T2W images, a similar tracing was used, and an EER>1.4 was used to indicate enhancement. Enhancement volumes, normalized as a percent of the total LA blood pool cavity volume, were calculated and compared.

### Statistical Analyses

Data analyses were performed using Excel (Microsoft 2010, Redmond, WA) or Stata/IC 10.0 (Stata Corporation, College Station, TX). Continuous variables are presented as mean ± one standard deviation. The relationship between in LGE and T2W enhancement was compared between time-points on a segment basis using Pearson’s chi-squared test. LA wall thickness comparisons were made using a two-tailed Student’s t-test. Linear regression was used to compare volumes of enhancement. Cohen’s kappa and intra-class correlation coefficient were used to characterize the reproducibility of categorical and ordinal measurements, respectively. A p value ≤0.05 was considered statistically significant, without Bonferroni correction.

## Results

Table 1 describes the 11/15 subjects who underwent post-PVI imaging. All available studies as described in Figure 1 were analyzed. Table 2 summarizes some of the results. The raw data supporting all results, and sample images, are provided as (Figures S1-S3, and Table S1).

**Table 1:** Patient characteristics and MRI findings for patients with post-PVI imaging.

**Patient Characteristic**	N = 11
Age (years)	52±11
Male gender, N (%)	9 (82%)
LV EF (%)*	56.0±9.3
LA dimension)	43±9
Ablation time (secs)	2900±1100
Paroxysmal AF, N (%)	8 (73%)
Anti-arrhythmic Medications, N (%)	10 (91%)
Hypertension, N (%)	5 (45%)
**LGE and T2W measurements**	All available
# segments enhanced (16 segment model)	
30-day LGE	13.7±1.8
24-hour LGE	13.6±1.1
24-hour T2W	12.1±3.2
Volume of enhancement (% of LA volume)	
30-day LGE	6.6±5.4
24-hour LGE	5.7±3.7
24-hour T2W	7.8±4.1

AF = atrial fibrillation, LA = left atrium, LV EF = left ventricular ejection fraction.

**Table 2:** Summary of Results.

	Pre-PVI	Post-PVI	P-value
LA wall thickness (mm)	7.0±1.8	10.7±4.1	0.038
Average EER	0.87±0.18	1.52±0.38	<0.001
% with 24-hour LGE	—	84%	
% with 24-hour T2W	8%	75%	
% with 30-day LGE	—	86%	
	**24-hour**	**30-day**	**p-value**
PV ostial LGE CNR	8.2±3.6	11.4±4.1	0.01
Valve LGE CNR	3.3±1.0	3.7±1.1	0.46
	**30-day LGE +**	**30-day LGE -**	**p-value**
24-hour EER	1.5±0.3	1.2±0.3	0.004
24-hour LGE CNR	7.3±3.3	3.0±5.8	0.037
	**Sensitivity** *	**Specificity** *	**Accuracy** *
24-hour EER>1.4	82% (75%–89% CI)	63% (54%–71% CI)	79%
24-hour LGE CNR>3.5	91% (86%–97% CI)	47% (40%–60% CI)	84%

* vs. 30-day LGE.

**Figure 4:**
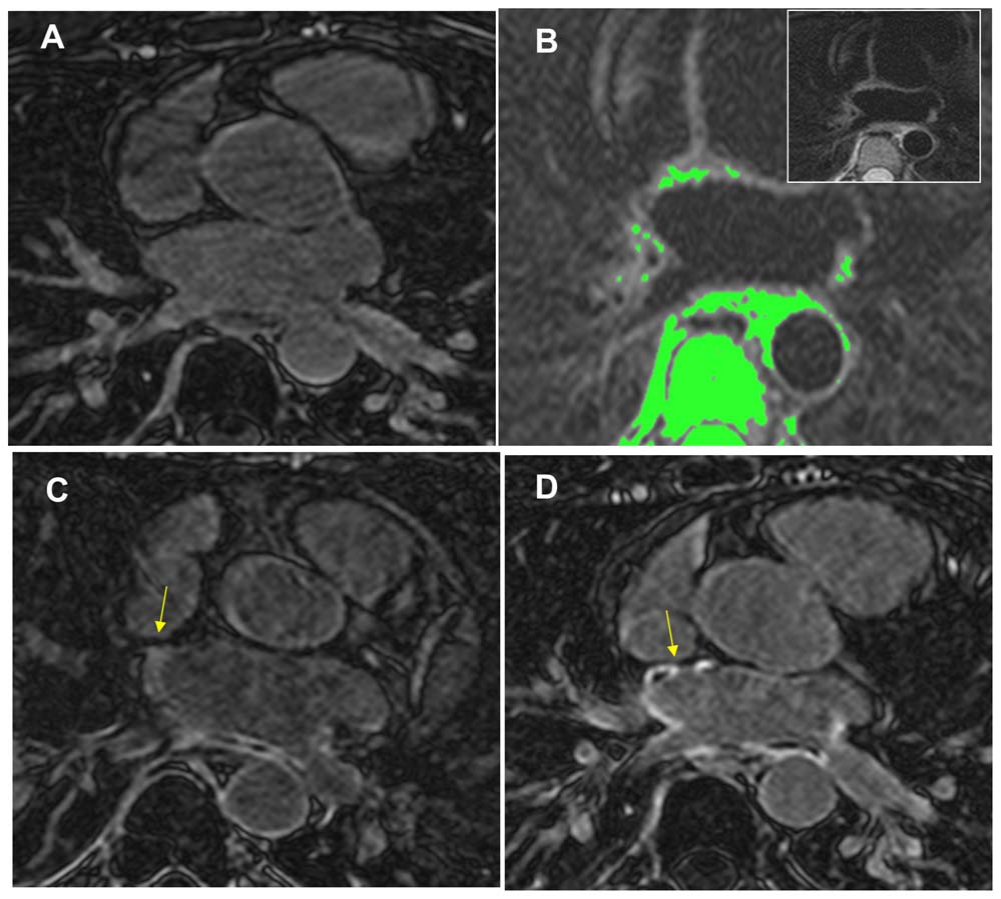
Axial CMR images demonstrating the progression of scar development of a single patient after PVI. A) Pre-PVI LGE scan shows no baseline scar. B) 24-hour post PVI T2W image showing areas of edema, highlighted in green, which had an EER>1.4. Inset shows original image. C) 24-hour LGE. D) 30-day post PVI LGE showing enhancement in the pulmonary veins which corresponded to 24-hour edema location. Note the less intense enhancement pattern of 24-hour LGE compared to 30-day LGE, with some enhancement not visible at 24-hours and dark “no-reflow “regions both early and late after PVI (yellow arrows).

Figure 4 shows matched slices at 3 time-points in a single subject. At 24-hours post PVI, T2W and LGE imaging display multiple areas of enhancement in the PV ostia and posterior LA wall indicating edema and injury. There was also visual correspondence between 30-day and 24-hour LGE, with a less intense pattern of enhancement on 24-hour LGE.

Figure 2G-I shows how matched locations were obtained in another subject, through registration. The registration achieved between 30-day LGE (blue shell) and 24-hour T2W (orange segmented pixels) was good. The correlation between the 24-hour T2W enhancement and 30-day LGE enhancement is demonstrated.

### LA Wall Thickness

The LA wall thickness, measured at the posterior wall of the RPSV, was increased by about 4 mm 24-hours after PVI (Table 2). Wall thickness measurement intraobserver variability was good: R = 0.88, and bias ± 2SDs of −0.4±2.2 mm, and intra-class correlation coefficient of 0.69 (strong agreement).

### Edema Enhancement Ratio: Pre- vs. 24-Hour Post-PVI

For pre-PVI AF patients, the average T2W EER of the LA wall to the LV myocardium was significantly lower than for 24-hour post-PVI patients (Table 2). The pre-PVI EER measurements were used to choose EER = 1.4 as the threshold for edema, using the mean +3SDs (0.87+3*0.18  = 1.41).

### CNR Comparison: 24-Hour vs. 30-Day LGE

The PV ostial CNR (LA wall to blood) of enhanced regions for 30-day LGE was greater than for 24-hour LGE (Table 2). Figure 2 and 4 demonstrate that some regions enhanced on 30-day LGE but not on 24-hour LGE (yellow arrows, Figure 4C,D), and that enhancement at 30 days had higher CNR, and better conspicuity. The CNR of the mitral valve was measured to be similar on 24 hour vs. 30 day LGE (Table 2).

### Correlation between 24-Hour T2W and LGE Findings and 30-day LA Wall LGE Enhancement at Matched Locations

The EER was higher in locations in which 30-day LGE enhancement developed compared to locations without later scar, as was the 24-hour LGE CNR (Table 2).

### Qualitative Assessment in 16 Segment Model

Pre-PVI, T2W imaging demonstrated 8% of PV quadrants were enhanced (>1.4 ER), with one-third of these regions located on the posterior wall of the right inferior PV (Figure 2C). At 24-hours post-PVI, 75% of quadrants had T2W enhancement and 84% of regions had findings on LGE. Agreement between 24-hour T2W enhancement and 24-hour LGE enhancement or MVO was found in 79% of quadrants, with a Kappa value of 0.37 (fair).

Ninety six quadrants (86%) were scarred by 30-day LGE. Using 30-day LGE as reference standard, the 24-hour T2W images had 82% sensitivity, 63% specificity, and an accuracy of 79% to predict 30-day LGE enhancement, on a per quadrant basis. The 24-hour LGE images had 91% sensitivity, 47% specificity and 84% accuracy. Agreement of 30 day LGE with acute LGE and T2W was significantly correlated by chi-squared test (p = 0.005 and p = 0.002, respectively). Table 2 summarizes these regional results.

Intraobserver agreement for qualitative assessments was 88% for T2W, 93% for 24-hour LGE, and 94% for 30-day LGE, with kappa values of 0.66, 0.68, and 0.71 for the three datasets, respectively (all substantial agreement).

### Volumetric Comparison of Enhancement on LGE and T2W

**Figure 5:**
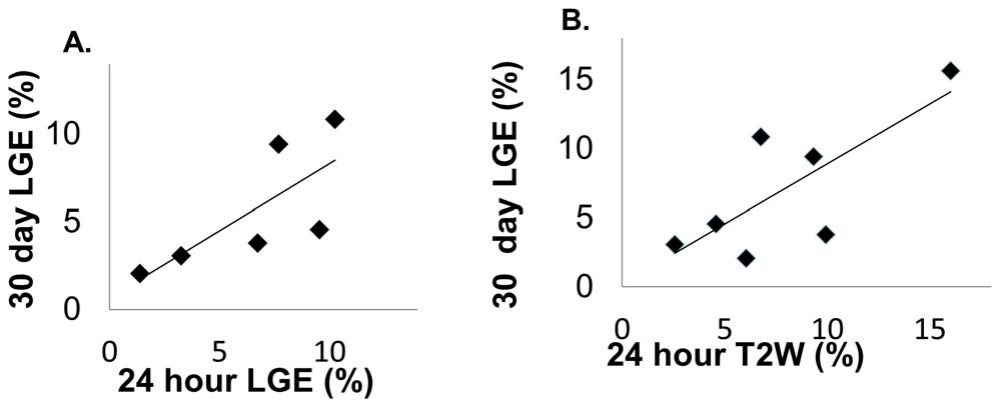
Linear correlations between volumes of LGE enhancement at 30 days, and volumes measured at 24-hours. A) 24-hour LGE vs. 30-day LGE (R = 0.76, p = 0.09). B) 24-hour T2W vs. 30-day LGE volume (R = 0.74, p = 0.04). All volumes of edema/injury were normalized by the patient’s LA volume.

30-day LGE enhancement volume significantly correlated with 24-hour edema (R = 0.76, p = 0.047), but did not correlate significantly for 24-hour LGE (R = 0.74, p = 0.092) (Figure 5).

## Discussion

In this prospective study of AF patients referred for their first PVI, we demonstrate the ability of CMR to visualize early edema and injury using T2W and LGE of the pulmonary veins and LA wall, confirming prior studies [16], [17]. Further, we demonstrate a correlation between patterns of injury on 24-hour imaging and 30-day LGE, a finding that has not been previously reported. The purpose of this study is to evaluate the feasibility of using acute CMR to predict chronic ablation patterns. The goal of PVI is to electrically isolate the PV by creating permanent scar around the pulmonary veins. Our study highlights that there is a relationship between early and later findings of post PVI injury, which is evident in the linear correlation between volume of scar on 30-day LGE, to volume of 24-hour edema, and the ability of LGE and T2W at 24-hours to predict later scarring. Whether the relationship is strong enough to provide peri-procedural guidance to improve ablation patterns is uncertain, since potentially even small gaps might result in PV reconnection [37], and since the accuracy of 24-hour imaging appears limited.

### Correlation of 24-Hour Enhancement with 30-Day in the LA and PVs

We found high sensitivity (91% and 82%) but lower specificity (47% and 63%) for both 24-hour LGE and 24-hour T2W imaging, respectively, for predicting LGE at 30 days. The low specificity suggests that some acute injury resolves. 24-hour T2W had greater specificity (fewer false positives) than LGE, which is perhaps most critical.

### Relationship to Prior Reports

This study found acute wall-thickening after PVI. Using electron beam computed tomography (CT), Okada et al. [23] reported the increased thickness of the right posterior wall after circumferential ablation was 5 mm, similar to our finding in the same location. Yokokawa et al. found an increase in LA roof thickness of 1–2 mm [24]. While our baseline measured wall thickness of 7 mm is greater than that reported by CT imaging (1–4 mm) [38], [39]. This is likely due to 5 mm slice thickness which could cause partial-voluming of the LA wall with other tissues, or increased apparent thickness due to the LA wall curvature. However, the relative change in thickness after PVI is similar to previous CT reports [23].

Badger et al. compared early LGE and very late LGE (3 months) and found no correlation, while early LGE appeared as a diffuse (i.e. wide-spread, low CNR) signal [17]. In agreement, we found that the CNR of the injured regions was higher on the 30-day vs. 24-hour LGE, despite that the valvular CNRs were not different. This is likely due to the very slow enhancement of acutely ablated tissue [10], requiring ∼45 minute to full enhancement, which is longer than the delay of 20–25 minutes used for LGE imaging [10]. Our study, in agreement with [26], found that 24-hour MVO regions developed into 30-day LGE enhancement (Figure 3); however, even at 30 days, MVO regions remained present and common (Figures 2–3). Finally, our study examined 24-hour T2W and correlated this with 30-day LGE, finding a significant relationship.

Regions with LGE and T2W edema spatially overlap in imaging ablation sites [10], [11]—this is known. An unanswered question is whether acute LGE or T2W is more predictive of later ablation patterns, and later outcome. Another question is whether some edema resolves without fibrosis development. Arujuna et al. [40] found evidence that acute edema without acute LGE indicates reversible injury, potentially predicting later recurrence. They found that patients with greater T2W edema ostial encirclement at an acute stage experienced AF recurrence, while those with more extensive LGE at the acute stage were free from AF at follow-up. They found widespread edema compared to LGE, acutely. Our studied differed from this prior study, in that we did not find that edema was more widespread than LGE, acutely. This difference could be explained by the use of TE = 60 ms and an EER threshold of 1.4 in our study, compared to use of a TE = 120 ms, and a edema threshold of 3 SDs above mean myocardial signal. Our threshold for edema was set more stringently, based on data from controls, and our TE was lower. We found that edematous regions with a greater EER were more likely to develop into LGE than regions with lower EER, i.e. evidence that minimal edema does not result in later fibrosis, agreeing with the prior work. Therefore, apparent differences—i.e. a good correlation of acute edema with later LGE in the present study—might be related to a higher threshold for edema in our study.

### Limitations

Our study population was small, mainly because recruitment of patients immediately after the PVI procedure is highly challenging. However, this study is unique in that both T2W and LGE images were available acutely, for comparison with 30-day LGE. T2W images were not routinely obtained prior to PVI for comparison with the 24-hour images, and therefore we could not perform a paired analysis of T2W findings pre and post-PVI. However, this cannot be expected to affect our findings that EER increases acutely after PVI, and that the LA wall thickens.

Our rigid registration of T2W and LGE images at 24-hours and 30-days may result in imperfectly matched locations for measuring and comparing signal intensity. Furthermore, the thicker slices used in T2W imaging is also a source of location mismatch. However, in spite of the possible mismatch, we found higher 24-hour T2W EER and LGE CNR in regions which exhibited 30-day LGE enhancement. Finally, it is possible that some enhancement on T2W is related to pericardial fluid-filled recesses [41], or diffuse inflammatory response associated with AF.

In conclusion, increased LA wall thickening and edema and injury are characterized on CMR early post-PVI with correlation to 30-day LGE scar.

## Supporting Information

Figure S1 24-hour LGE patient image.
(JPG)

Figure S2 30-day LGE patient image.
(JPG)

Figure S3 24-hour T2W patient image.
(JPG)

Table S1 Raw measurements. A) Data of Figure 4. B) Data on regional assessment of injury using 16 segment model. C) CNR and EER measurements.
(XLSX)
