# Supplementary figures and images for: Cardiovascular Magnetic Resonance Imaging of Scar Development Following Pulmonary Vein Isolation: A Prospective Study
Source: PLoS One. 2014 Sep 24;9(9):e104844. doi: 10.1371/journal.pone.0104844 (PMC4174508; doi:10.1371/journal.pone.0104844)

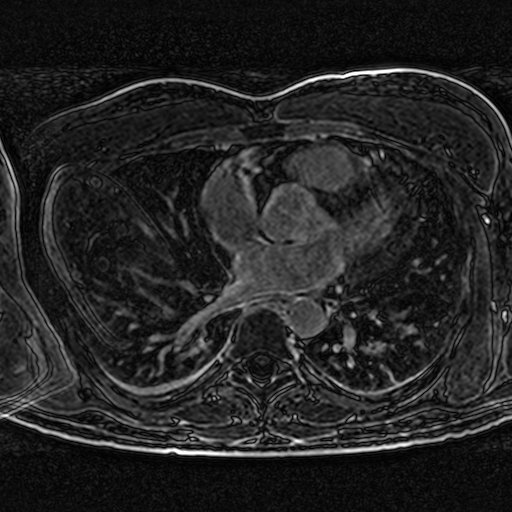

Supplement: Figure S1 — 24-hour LGE patient image. (JPG) [file pone.0104844.s001.jpg]

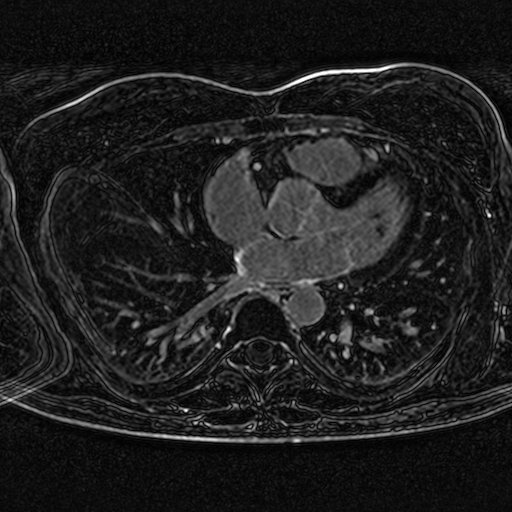

Supplement: Figure S2 — 30-day LGE patient image. (JPG) [file pone.0104844.s002.jpg]

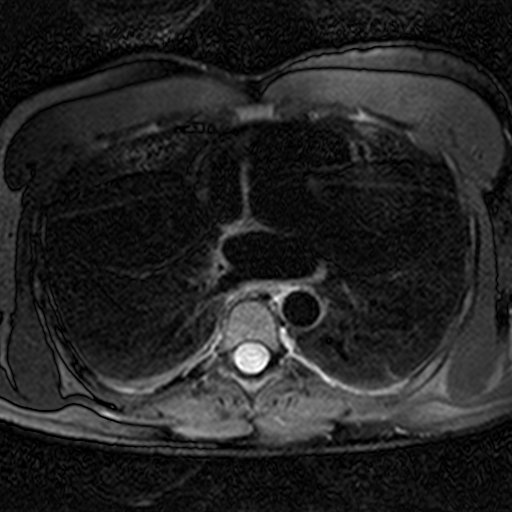

Supplement: Figure S3 — 24-hour T2W patient image. (JPG) [file pone.0104844.s003.jpg]
